# Anticancer Activities of Ricin-Liposome Complexes on SKMEL-28 Cells

**DOI:** 10.31557/APJCP.2019.20.7.2117

**Published:** 2019

**Authors:** Nguyen Thi Bich Loan, Ngo Ngoc Trung, Nguyen Thi Le Na, Nguyen Dinh Thang

**Affiliations:** 1 *Department of Biochemistry and Molecular Biology, *; 4 *The Key Laboratory of Enzyme and Protein Technology, VNU University of Science, Vietnam National University, *; 3 *Institute of Chemistry and Environment, High Command of Chemistry, Ministry of National Defense, Vietnam,*; 2 *Unit of Therapeutic Chemistry and Pharmacognosy, University of Mons (UMONS), Belgium. *

**Keywords:** ricin- migration- invasion- tumorigenesis- cytotoxicity- melanoma- cancer

## Abstract

**Background:**

Ricin has been reported as a potential chemical for cancer treatment. However, so far, the application of ricin in cancer treatment is very limited because of its non-specificity. Methods: In this study, ricin were conjugated/encapsulated with DOTAP/DOPE liposome to form ricin-liposome complexes (ricin-lipososme1, ricin-liposome2, ricin-liposome3 and ricin-liposome4). Characteristics of ricin-liposome complexes were analyzed and their effects on survival, apoptosis, migration, invasion and tumor formation of SKMEL-28 melanoma cells were examined by carrying out the MTT assay, apoptosis assay, scratch wound healing assay, invasion assay and soft-agar colony formation assay, respectively.

**Results:**

Ricin-liposome complexes had even size-distribution with average size of around 340 nm. These ricin-liposome complexes were able to penetrate into the cells via endocytosis with the highest ability of the ricin-liposome3. It also showed that ricin-liposome3 expressed very high toxicity with the IC50 of 62.4 ng/mL and followed by ricin-liposome4 (286.4 mg/mL), ricin-liposome2 (417.5 ng/mL), and ricin-liposome1 (604.3 ng/mL) to SKMEL-28 cells at 36 hours post treatment. At the concentrations of IC10 (10.1 ng/mL), ricin-liposome3 strongly induced necrosis and apoptosis of SKMEL-28 cells up to 25.6% and 11.4%, respectively. Moreover, ricin-liposome3 expressed great anticancer properties by decreasing the migration, invasion and tumor formation abilities of SKMEL-28 cells of 7.5 folds, 4.3 folds and 5.9 folds, respectively, compared with those of control SKMEL-28 cells.

**Conclusion:**

The obtained results from our study suggest that although ricin is listed as one of the most poisonous substances in nature, it can be used in the complex forms with liposome to increase its specificity to apply in treatment of melanoma and other cancers.

## Introduction

Ricin, a dominant chemical in seeds of castor bean, is a glycoprotein of the lectin family (Marwat et al., 2017; Kumar et al., 2004). Ricin consists of two polypeptide chains with total molecular weight of 64. It is considered as one of the most poisonous substances in nature and great toxic for humans (Funatsu et al., 1971). Because of its toxicity, ricin can be used for bioterrorism. Ricin can penetrate into the cell through its high affinity to galatose residues on the cell surface and cause cell death (Funatsu et al., 1971; Michael et al., 1997; Kumar et al., 2004; Suresh et al., 2007). Ricin has also been reported as a strong immunotoxin for human and animal cells (Engert et al., 1990; Engert et al., 1994; Weidle et al., 2014).

In a contrast way, ricin has been reported that it can be used to treat various cancers, including leukemia, breast cancer, cevical cancer and melanoma (Spitler et al., 1986; Spitler et al., 1987; LoRusso et al., 1995; Tyagi et al., 2013; Tyagi et al., 2015; Polito et al., 2016 and Li et al., 2017). Recently, ricin has been studied to encapsulate into polymer and/or liposome to decrease its side-effect toxic toward normal cells to increase its benifits in cancer treatment (Epler et al., 2012; Tyagi et al., 2013; Tyagi et al., 2015). 

Since melanoma is an aggressive cancer with high metastatic ability, the increase in incidence is a threat to public health (Gray-Schopfer et al., 2007; Chen et al., 2016). Therefore, it is important to find new effective therapies to treat melanoma. Although ricin has potential as an anticancer agent, its specificity is one of the greatest challenges for its applications in cancer therapy. Ricin can conjugate with antibody to form an immunotoxin for melanoma treatment (Tyagi et al., 2013; Tyagi et al., 2015). Recently, developments of encapsulation system such as polymer and liposome have been considered as effective methods for drug delivery to target cancer cells. 

Although ricin can be used as a drug for cancer treatment, it is impossible to purchase from commercial sources because of its poisonous property and potential application for bioterrorism. Therefore, previous study we had succeeded in extracting, isolating and purifying ricin from seeds of castor been (Ngo et al., 2016). In this study, we have concentrated in making liposomal ricin and investigating their anticancer activities on SKMel-28 melanoma cells. 

## Materials and Methods

*Liposome vesicle preparation*

The DOTAP/DOPE liposome vesicles were made following the previous report (Kim et al., 2015). The neutral lipid DOPE (1,2-dioleoyl-sn-glycero-3-phosphoethanolamine) and the cationic lipid DOTAP (N-[1-(2,3dioleoyloxy)propyl]-N,N,N-trimethylammonium chloride) (Sigma, USA) were dissolved in chloroform. The lipid solutions were mixed at weight ratio of 1:1 with different weight/weight (mg/mg) of DOPE/DOTAP, including: 0.5/05, 1/1, 2.5/2.5 and 5/5. Chloroform was evaporated under vacuum overnight to make the lipid ﬁlms. Then, the appropriate amount of ricin was added to dried lipid ﬁlms and the solutions were incubated at 4oC overnight. Then, the complexes were vortexed for 3 minutes and sonicated for 20 minutes at 40oC to form unilamellar ricin-liposome vesicles. The obtained ricin-liposome complexes were stored at 4oC. To test the efficiency of endocytosis of liposome vesicles into the cells, instead of ricin, Green Flourescent Protein (GFP) was used to make GFP-liposome complexes. 

*Cell culture*

The malignant melanoma SKMEL-28 cells were purchased from ATCC, United State. Cells were cultured in RPMI supplemented with penicillin (400 U/mL), streptomycin (50 mg/mL), L-glutamine (300 mg/mL) and 10% fetal bovine serum (FBS; Sigma, Deisenhofen, Germany) under a humidified atmosphere of 5% CO_2_ at 37^o^C. 

*MTT assay*

MTT assays were carried out following protocol of the KIT (Vybrant MTT Cell Proliferation Assay Kit of Thermo Fisher Scientific, United State) to test the cytotoxicity. Briefly, 1×10^4^ SKMEL-28 cells were seeded into the 96-well plate and treated with ricin-liposome for 36 hours. Cells were then washed and treated with 10 µL of the 12 mM MTT stock solution to each well and incubated at 37°C for 4 hours before adding 100 µL of the SDS-HCl solution to each well and mixed thoroughly using the pipette. Next, cells in the microplate were incubated at 37°C for 4 hours in a humidified chamber before mixing and reading the absorbance at 570 nm.

*Apoptosis assay*

Cells were seeded at 30 to 40% confluence in 6-cm plates and incubated at 37°C for overnight, medium was aspirated and replaced with fresh medium and treated with or without ricin-liposome for 36 hours. Cells were washed with PBS before collecting and then re-suspending in annexin binding buffer (Life technology) at 1×10^6^ cells/ml. Cells were stained with Propidium Iodide-PE (PI-PE) and Annexin V-FITC according to the manufacturer’s protocol and assayed on a FACSCanto II (BD Biosciences). The percentage of apoptotic cells was measured as the percentage of annexin V-positive cells. 

*Scratch wound healing migration assay*

Cell wound healing was performed, as described previously (Thang et al., 2011). Cells seeded in six-well plates were incubated overnight in 1 ml RPMI-1640 medium. The cells were grown to 100% confluence on the plates in RPMI-1640 medium supplemented with 10% FBS. Scratch wounds were created in confluent monolayers using a sterile p200 pipette tip. A total of four perpendicular semi-opaque marks were placed across each scratch on the external surface of the well to standardize quantitative analysis. Following washing, the suspended cells were washed three times, and the wounded monolayers were again cultured in RPMI-1640 medium. Following incubation for 6 hours and 22 hours, repopulation of the wounded areas was observed under phase-contrast microscopy. The size of the scratch wound area was determined at each time point.

*In vitro tumorigenesis assay*

A colony formation assay was performed to assess the development of tumor in vitro. Anchorage-independent growth was evaluated by the soft-agar colony formation assay according to the method reported in the previous study (Thang et al., 2011). Cells were treated with or without ricin-liposome and incubated at 37°C for 36 hours. Then, 2×104 cells were mixed with 2 mL of 0.36% soft agar in RPMI medium, poured onto slightly solid 0.72% hard agar in medium, and cultured for 4 weeks. Colonies formed in the soft-agar were observed under microscope and colonies exceeding 50 µm in diameter were counted and presented. 

*Invasion assay*

Cell invasion ability was evaluated by an invasion assay according to previous report (Thang et al., 2011). Briefly, cells were pre-treated with or without ricin-liposome for 36 hours. Then, 2×10^5^ cells in 300 ml starving medium (0.5% FBS) were applied into a matrigel-coated upper chamber of 8 mm in diameter (8 mm in pore size). Then the upper chambers were placed in 24-well culture plates containing 600 ml conditioned medium with 0.5% FBS to trigger invasion activity. Cells were incubated for 12 hours and then invading cells on the bottom of the chamber were fixed with formalin 10%, stained with crystal violet and counted under a microscope.

*Statistical analysis*

Results from three independent experiments in each group were statistically analyzed by Student’s t-test. The SPSS (version 18) software package (SPSS Japan Inc.) was used for statistical analyses, and the significance level was set at p < 0.05. 

## Results

*Creation of ricin-liposome complexes*

**Figure 1:**
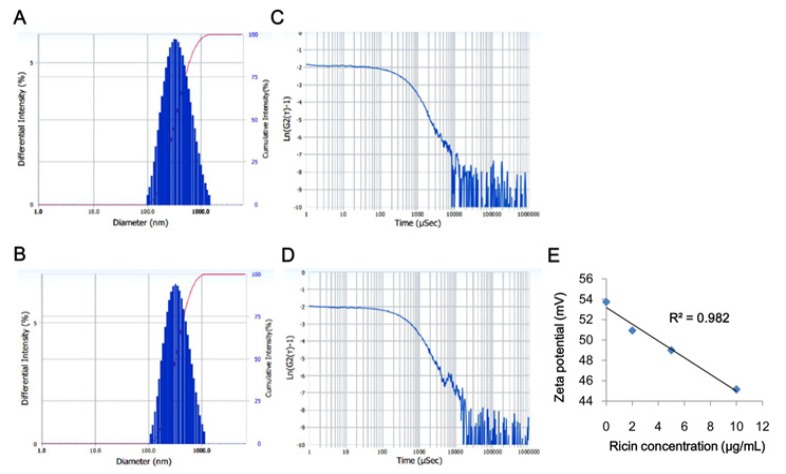
Characteristics of Ricin-Liposome Vesicles. The size distributions of control DOTAP/DOPE liposome (A and C) and ricin-DOTAP/DOPE liposome (B and D); The inverse-linear correlation between zeta potential of ricin-liposome and concentration of ricin (E)

Ricin-liposome complexes were made with different formulas of DOTAP/DOPE and presented as ricin-liposome1 for DOTAP/DOPE/ricin of 0.5/0.5/2.5 (mg/mg/μg), ricin-liposome2 for DOTAP/DOPE/ricin of 1.0/1.0/2.5 (mg/mg/μg), ricin-liposome3 for DOTAP/DOPE/ricin of 2.5/2.5/2.5 (mg/mg/μg) and ricin-liposome 4 for DOTAP/DOPE/ricin of 5.0/5.0/2.5 (mg/mg/μg). Size distribution of ricin-liposome vesicles in solution were measured and presented in the Figure 1. The control liposome (without ricin added) had an average size of 337 nm (Figure 1 A and C), while the ricin-liposome complexes had an average size of 351 nm (Figure 1 B and D). This meant that that ricin might be conjugated with liposome to increase the size of the complexes. The DOPE/DOTAP liposomes were cationic liposomes, and protein always had negative charge on its surface; therefore, beside the encapsulation into the liposome, ricin might form electrostatic interaction with liposome to form ricin-liposome complexes. For sure that all the added ricin were encapsulated/conjugated with liposome, zeta potentials of these complexes were measured. It showed that adding ricin at different concentrations of ricin (0, 2.5, 5 and 10 μg) to DOTAP/DOPE (2.5mg/2.5mg), the zeta potentials were gradually decreased with an inverse-linear model with R2 value of 0.982 (Figure 1E). 

*Endocytosis of protein-liposome into the cultured cancer cells*

**Figure 2:**
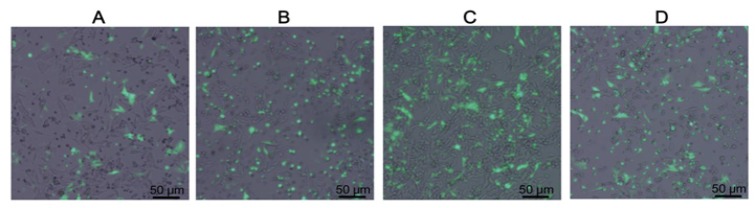
Transfection Efficiency of GFP-DOTAP/DOPE Liposome into SKMEL-28 Cells. The transfection abilities (fluorescent signal) of various formulas GFP-liposomes, including: GFP-liposome1 for DOTAP/DOPE/GFP of 0.5/0.5/2.5 (mg/mg/μg) (A), GFP-liposome2 for DOTAP/DOPE/GFP of 1.0/1.0/2.5 (mg/mg/μg) (B), GFP-liposome3 for DOTAP/DOPE/GFP of 2.5/2.5/2.5 (mg/mg/μg) (C) and GFP-liposome4 for DOTAP/DOPE/GFP of 5.0/5.0/2.5 (mg/mg/μg) (D)

Ricin affected on cells via inactivating of ribosome, therefore, the transportation of liposome into the cells played very important role. To test the endocytosis efficacy of protein-liposome complexes into SKMEL-28 cells, instead of ricin-liposomes, GFP-liposomes including GFP-liposome1 for DOTAP/DOPE/GFP of 0.5/0.5/2.5 (mg/mg/μg), GFP-liposome2 for DOTAP/DOPE/GFP of 1.0/1.0/2.5 (mg/mg/μg), GFP-liposome3 for DOTAP/DOPE/GFP of 2.5/2.5/2.5 (mg/mg/μg) and GFP-liposome4 for DOTAP/DOPE/GFP of 5.0/5.0/2.5 (mg/mg/μg) were added into SKMEL-28 cultured cells. The pictures of cells were captured under fluorescent microscope after incubation for 36 hours. The obtained results revealed that the GFP-liposome3 had the highest endocytosis efficiency into the SKMEL-28 cells, following by GFP-liposome4, GFP-liposome2 and GFP-liposome1 (Figure 2).

**Figure 3:**
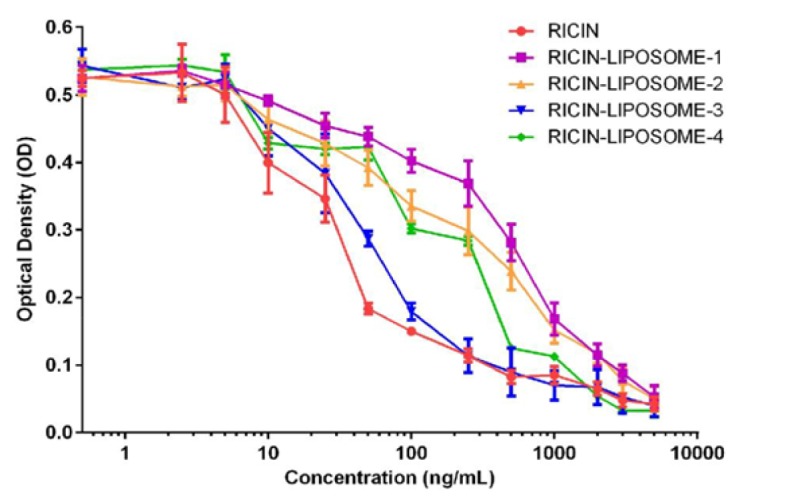
Cytotoxicity of Ricin-Liposome on SKMEL-28 Cells. The toxicities of free ricin, ricin-liposome1, ricin-liposome2, ricin-liposome3 and ricin-liposome4 at different concentrations (0.1, 0.5, 1.0, 2.5, 5.0, 10, 100, 250, 500, 1,000, 2,000 and 5,000 ng/mL) on SKMEL-28 cells after 36 hrs incubation were presented

**Figure 4:**
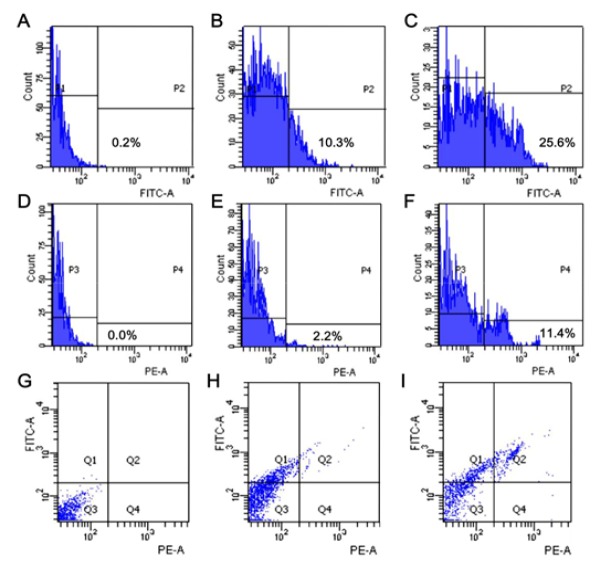
Effects of Ricin-Liposome on Necrosis and Apoptosis of SKMEL-28 Cells. Necrotic percentage (P2) of SKMEL-28 cells without any treatment as negative control (A), ricin-liposome2 treatment (B) and ricin-liposome3 treatment (C). Apoptotic percentage (P4) of SKMEL-28 cells without any treatment as negative control (D), ricin-liposome2 treatment (E) and ricin-liposome3 treatment (F). Dot blot map presented the percentage of cells at different stages of early apoptosis (Q2), late apoptosis (Q4), normal (Q3) and necrosis (Q1) for negative control (G), ricin-liposome2 treatment (H) and ricin-liposome3 treatment (I)

**Figure 5:**
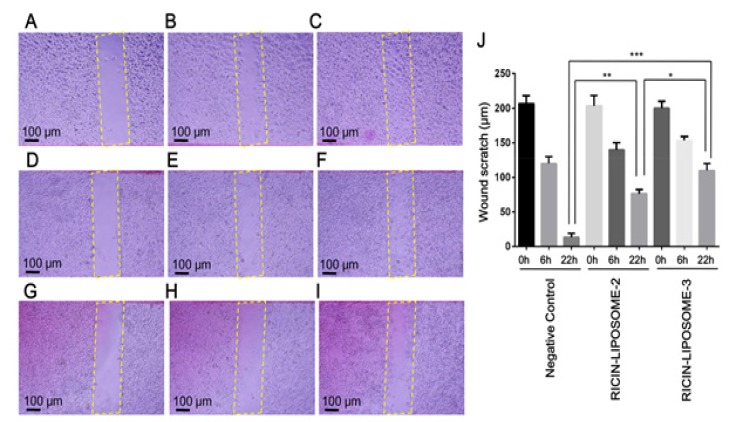
Effects of Ricin-Liposome Complexes on Migration Activity of SKMEL-28 Cells. Wound healing activities of control cells, ricin-liposome2 treated cells and ricin-liposome3 treated cells at 0h (A, D, G), 6h (B, E, H) and 22h (C, F, I) post wound-creating were presented respectively. The differences in migration abilities of control cells and ricin-liposome treated cells were presented in a graph (J). *, ** and ***, significant differences from control with P < 0.05, P < 0.01 and P < 0.001 by Student’s t-test, respectively

**Figure 6:**
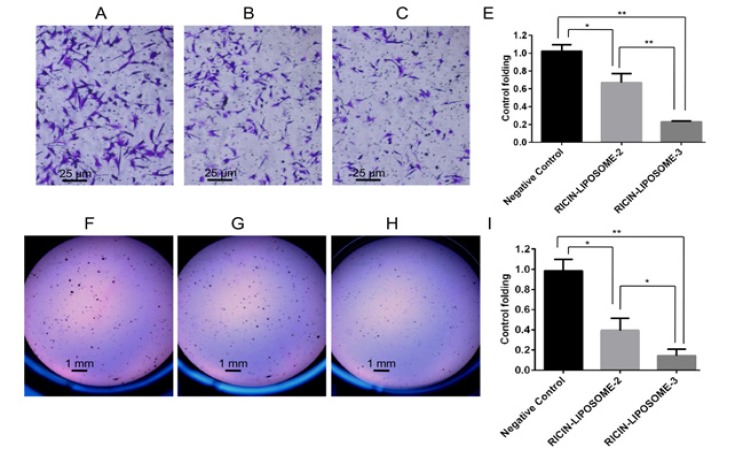
Effects of Ricin-Liposome Complexes on Invasion and Tumor Formation of SKMEL-28 Cells. Invasion activities of control cells (A), ricin-liposome2 treated cells (B) and ricin-liposome3 (C) and the differences in invasion abilities of control cells and ricin-liposome treated cells (D) were presented. Tumor formation activities of control cells (E), ricin-liposome2 treated cells (F) and ricin-liposome3 (G) and the differences in tumor formation abilities of control cells and ricin-liposome treated cells (H) were presented. * and **, significant differences from control with P < 0.05 and P < 0.01 by Student’s t-test, respectively

*Toxicity of ricin-liposome complexes to the cultured cancer cells*

The cytotoxicity of ricin-liposome complexes were investigated and presented in the Figure 3. The SKMEL-28 was treated with complexes containing of ricin at different concentrations of 0.5, 1, 2.5, 5, 10, 25, 50, 100, 250, 500, 1,000, 2,000, 5,000 ng/mL. The obtained results showed that the IC50/IC10 values were 604.3/25.5 ng/mL, 417.5/14.8 ng/mL, 62.4/10.1 ng/mL and 286.4/10.7 ng/mL for ricin-liposome1, ricin-liposome2, ricin-liposome3 and ricin-liposome4, respectively; while the IC50/IC10 of ricin in free form was 36.2/7.5 ng/mL (Figure 3). This meant that ricin in the complex with liposome were lower toxic compared with free ricin. However, the toxicity of ricin-liposomes, especially ricin-liposome3, was still high enough to kill cancer cells. Further, ricin-liposome2 and ricin-liposome3 were used to investigate their effects on necrosis and apoptosis of SKMEL-28 cells. SKMEL-28 cells were treated with ricin-liposome2 at concentration of 15 ng/mL (IC10) and ricin-liposome3 at concentration of 10 mg/mL (IC10) for 36 hours before collecting for the apoptosis analysis by annexin V apoptosis assay on FACSCanto system. It indicated that ricin-liposomes induced both necrosis and apoptosis of SKMEL-28 cells. The percentages of cells in necrosis stage were 0.2%, 10.3% and 25.6% in the negative control, ricin-liposome2 and ricin-liposome3 treatments, respectively (Figure 4A-B-C). And, the dot-blot map presented the percentage of cells at different stages of early apoptosis (Q2), late apoptosis (Q4), normal (Q3) and necrosis (Q1) (Figure 4-G-H-I).

*Anticancer effects of ricin-liposomes on the cultured cancer cells*

Further, the effects of ricin-liposome complexes on migration, invasion and tumor formation, which are hallmarks for cancer characteristics of cancer cells, of SKMEL-28 cells were investigated by performing the scratch wound healing assay, invasion assay and soft-agar colony formation assay. The scratch areas were measured at starting point (0h) (Figure 5 A, D, G), after 6 hours (6h) (Figure 5 B, E, H) and after 22 hours (22h) (Figure 5 C, F, I). It showed that, at six hours post wound creating, migration ability of cells had stated but there was slight difference between ricin-liposome treated cells and negative control cells (Figure 5-B-E-H, and J). However, at the 22 hours post wound making, the migration activity of the cells with ricin-liposome2 and ricin-liposome3 treatments were only about 1/4.6 and 1/7.5 compared with that of negative control cells, respectively (Figure 5C, F, I, J). More importantly, ricin-liposome also inhibited invasion activity and tumorigenesis of SKMEL-28 cells (Figure 6). Treatments with ricin-liposome2 and ricin-liposome3 decreased invasion activity of SKMEL-28 1.5 folds and 4.3 folds compared with that of control SKMEL-28 cells, respectively (Figure 6 A, B, C, D). And, treatments with ricin-liposome2 and ricin-liposome3 also caused to decrease of tumor formation ability of SKMEL-28 cells 2.3 folds and 5.9 folds when comparing with that of control SKMEL-28 cells, respectively (Figure 6 E, F, G, H).

## Discussion

Ricin is comprised of a catalytic A subunit and a lectin B subunit (Ready et al., 1986). Ricin expresses the toxicity to human and animal cells via its ability to chemically inactivate the eukaryotic ribosome and finally results in cell and/or tissue death (Endo et al., 1987 and Endo et al., 1988). Although ricin is a very toxic chemical, it has also been reported as a potential drug for treatments of different cancers, including leukemia, breast cancer, bladder cancer, cervical cancer and melanoma (Spitler et al., 1987; LoRusso et al., 1995; Tyagi et al., 2013; Sadraeian et al., 2013; Tyagi et al., 2015; Polito et al., 2016; Li et al., 2017).

These days, chemicals extracted from medicinal plants have been concentrated to find out new drugs for cancer treatment (Yang et al., 2018; Phan et al., 2018). Ricin is a protein extracted from seed of castor beans (Ngo et al., 2016). The application of ricin in cancer treatment is limited because of its non-specificity. Therefore, recently, ricin was used in monoclonal antibody-conjugated forms to increase its specificity (Epler et al., 2012; Alipour et al., 2013; Buonocore et al., 2011). More importantly, developments of encapsulation system such as polymer and liposome have now been effectively used for delivering of drug to the target cells. Several previous studies reported that ricin-encapsulated liposomes showed antitumor abilities in various types of cancers (Tyagi et al., 2013; Epler et al., 2012; Buonocore et al., 2011). 

In this study, for the first time, the complexes of ricin and DOTAP/DOPE liposome were made and the properties of these complexes were examined. The results indicated that the ricin-liposome complexes had an uniform distribution in size. The potential zeta of the complexes depended on the concentration of ricin added. At the tested concentrations, all the ricin was conjugated/encapsulated with liposome. The cytotoxicity tests indicated that although the toxicities of ricin-liposomes complexes toward SKMEL-28 cells were lower than that of free ricin, the IC50 values were very low. This means that ricin-liposome complexes were very high toxic for killing of cancer cells. In addition, the toxicity of the ricin-liposoome complexes relied on the transfection efficiency into the cell via endocytosis. And the transfection efficiency, in turn, depended on the liposome formulas (Kim et al., 2015). In this study, our results showed that the liposome with the formula of DOPE/DOTAP:2.5/2.5 had the highest transfection efficiency for entering into the cell. In consistence with that, the ricin-liposome complex made from this formula (ricin-liposome3) was the most toxic substance to the SKMEL-28 cells. More importantly, ricin-liposomes complexes at low concentrations of IC10 (10 – 15 ng/mL) could significantly inhibit the migration, invasion and tumorigenesis, which are hallmarks of cancer cells (Thang et al., 2011; Tyagi et al., 2013; Phan et al., 2018), of SKMEL-28 melanoma cells in-vitro expresssing via the scratch wound healing assay, invasion assay and soft agar colony formation assay, respectively.

This study suggested that DOTAP/DOPE liposome can be used to load ricin protein and these ricin-liposome complexes may be additionally modified by conjugating with driving molecules to attach to their corresponding receptors harboring on the target cells for further applications in targeting treatment of cancers. 
